# Interaction between nematodes and bacteria enhances soil carbon sequestration under organic material amendments

**DOI:** 10.3389/fmicb.2023.1155088

**Published:** 2023-05-12

**Authors:** Guangping Shi, Lu Luan, Guofan Zhu, Zhaoyang Zeng, Jie Zheng, Yue Shi, Bo Sun, Yuji Jiang

**Affiliations:** ^1^State Key Laboratory of Soil and Sustainable Agriculture, Institute of Soil Science, Chinese Academy of Sciences, Nanjing, China; ^2^University of Chinese Academy of Sciences, Beijing, China; ^3^Ecological Experimental Station of Red Soil, Chinese Academy of Sciences, Yingtan, China; ^4^College of Land Resources and Environment, Jiangxi Agricultural University, Nanchang, China

**Keywords:** bacterial community, nematode assemblage, nematode indices, carbon mineralization, microbial metabolic quotient, organic material amendments

## Abstract

The process of carbon (C) sequestration plays an important role in soil fertility and productivity, yet most studies have focused on the individual role of the bacterial community. However, an in-depth mechanistic understanding of how soil nematodes interact with the bacterial community to regulate soil C accumulation is still lacking. We conducted a 10-year field experiment to explore the nematode and bacterial communities and determine the influence of nematode-bacteria interactions on C mineralization, microbial metabolic quotient (*q*CO_2_), and carbon use efficiency (CUE) under the organic material amendments, including chemical fertilizers with straw (NS), chemical fertilizers with straw and pig manure (NSM), and chemical fertilizer with straw biochar (NB). Here, our results showed the abundance of bacterial and nematode communities was significantly higher under NS, NSM, and NB treatments than under chemical fertilizers (N) treatment, with the highest abundance under the NSM treatment. The enrichment index and functional dispersion index were significantly higher under NSM treatment than under N, NS, and NB treatments, while the channel index followed the opposite pattern. Structural equation modeling indicated that the potential predation pressure induced by nematodes may improve bacterial abundance, with positive cascading effects on C sequestration. Collectively, our study highlights the functional importance of nematode-microorganism interactions in mediating C dynamics under organic material amendments.

## Introduction

Soil organic carbon (SOC) reservoir is the major component of the carbon (C) pool in terrestrial ecosystems and plays an important role in soil fertility and crop yield (Cook-Patton et al., 2020). Excessive use of chemical fertilizers degrades soil structure and reduces C content, while the application of organic materials, especially straw and manure, is considered an effective measure to improve soil quality and C sequestration (Wei et al., 2021). Straw itself is rich in mineral elements and nutrients and is often combined with chemical fertilizers to simultaneously improve C turnover and accumulation (Berhane et al., 2020). Pig manure efficiently increases easily decomposable particulate organic carbon due to its high nitrogen content and low carbon to nitrogen ratio (Gross and Glaser, 2021). A comprehensive understanding of the biological mechanisms of C turnover is paramount to quantifying C accumulation potential in agricultural ecosystems.

Microorganisms are the primary C decomposers in agricultural ecosystems (Zechmeister-Boltenstern et al., 2015). External carbon sources cause a series of changes in the abundance and composition of the soil microbial community, thereby affecting microbial metabolic quotient and C mineralization. Microbial biomass carbon is an essential component of the C pool (Fan et al., 2021). Microorganisms alter the amount and chemical composition of soil organic matter through decomposition, respiration, growth, and death (Min et al., 2021). While C measurements can represent changes in the magnitude of C storage, indicators related to microbial biomass and respiration can predict long-term trends in C sequestration (Zhou et al., 2017). The ratio of soil microbial respiration to growth (*q*CO_2_) indicates the energy required to maintain microbial biomass (Fließbach et al., 2000), with the higher *q*CO_2_ indicating faster nutrient turnover and lower carbon use efficiency (CUE) (Wardle and Ghani, 1995). It is generally accepted that increasing microbial CUE can enhance C sequestration (Sinsabaugh et al., 2013). However, the potential mechanism by which CUE varies with substrate quality and predation pressure needs to be further investigated (Dijkstra et al., 2015; Kane et al., 2022).

Traditional research has mainly focused on the individual role of the soil microbial community in C turnover and accumulation, but the understanding of how the interactions between soil animals and microorganisms affect C dynamics under the organic material amendments is still emerging (Van Den Hoogen et al., 2019). Nematodes are widely known as the most abundant soil invertebrates and contribute significantly to C decomposition and cycling through their interactions with the bacterial community (Jiang et al., 2013; Pausch et al., 2016; Zhang et al., 2021). Nematode predation alters the diversity and structure of the bacterial community and limits bacterial community size and physiology, thereby affecting C-related microbial functions (Grandy et al., 2016; Wang et al., 2021) A global-scale study further highlights the importance of nematode assemblages in soil food webs, nutrient cycling, and ecosystem functioning (Van Den Hoogen et al., 2019). Based on their board distribution and feeding traits, the ecological indices of nematode communities are well established as indicators of soil environmental conditions and food web status (Ferris et al., 2001). Although a rapidly growing literature has investigated the nematode-microbe interactions, there is still insufficient research on the direction and strength of nematode-microbe interactions on the regulation of C dynamics at different fertility levels (Grandy et al., 2016). Therefore, it is necessary to explore the role of nematodes on microorganisms to better understand the ecological role of nematode predation in mediating C dynamics.

Here, we aimed to examine how the influence of nematode predation affects C mineralization and sequestration by affecting the bacterial community in response to the organic material amendments. We used a field experiment under five fertilization treatments, and focused on answering the following two questions: (1) how the diversity, abundance, and composition of bacterial and nematode communities varied with the addition of different organic materials? and (2) whether and to what extent the nematode-bacteria interactions mediated C sequestration subject to organic material amendments? To address these questions, we examined the bacterial community structure by using Illumina sequencing, identified nematodes under a microscope, and determined *q*CO_2_ and CUE. We hypothesized that the nematode predation on bacterial community would improve C turnover and accumulation under organic material amendments.

## Materials and methods

### Description of the field experiment

The long-term experiment was established at the Yingtan National Agro-Ecosystem Observation and Research Station of the Chinese Academy of Sciences, Jiangxi Province (E116°55′30″, N28°15′20″). The climate of the region is characterized as warm and monsoon with a mean annual temperature of 17.6°C and a mean annual precipitation of 1795 mm. The soil is an acid loamy clay derived from Ferric Acrisols in the FAO classification system and Udic Ferralsols in the Chinese Soil Taxonomy.

The field experiment was initiated in 2011, and included five treatments with three replications in a randomized design: (1) no fertilizer (CK); (2) NPK chemical fertilizers (N); (3) NPK chemical fertilizers with straw (NS); (4) NPK chemical fertilizers with straw and pig manure (NSM); and (5) NPK chemical fertilizers with straw biochar (NB). Each of the 15 plots was applied to 20 m long and 5 m wide. The monoculture corn (cultivar Suyu 24) was annually planted in April and harvested in July. Except for CK, urea (N 150 kg hm^−2^, Shanxi Weihe Heavy Chemical Co., Ltd.), calcium–magnesium-phosphate fertilizer (P_2_O_5_ 75 kg hm^−2^, Yifeng Fertilizer Co. Ltd.), and potassium fertilizer (K_2_O 60 kg hm^−2^, Mosaic Potash Esterhazy LP.) were once applied in each treatment. The amount of corn straw, pig manure, and straw biochar was applied as 1,000 kg C hm^−2^ year^−1^.

### Soil sampling and chemical analysis

Soil samples were collected after the corn harvest at the end of July 2020. A total of 10 sampling points were taken from 0 to 20 cm topsoil samples using an S-shaped sampling method, and 1 kg soil samples were taken using the quartering method after evenly mixing. After that, fresh soil samples were placed in clean bags on ice and immediately transported to the laboratory. Soil samples in each treatment were separated into two subsamples for analysis of soil chemical properties, and the bacterial and nematode communities, respectively.

Soil pH was determined using a glass electrode at a water: soil ratio of 2.5:1 (v/w). Soil organic carbon (SOC) and total potassium (TK) were measured by the Walkley-Black wet digestion method and flame photometry, respectively (Jackson, 1958). Total phosphorus (TP) and total nitrogen (TN) were determined by molybdenum-blue colorimetry and Kjeldahl digestion, respectively (Zheng et al., 2009). Cation exchange capacity (CEC) was determined by the ammonium acetate saturation method (Kitsopoulos, 1999). Microbial biomass carbon (MBC) was measured by the chloroform fumigation incubation method (Vance et al., 1987).

### Quantitative PCR and illumina sequencing

The DNA from the soil sample was extracted following standard procedures of the DNeasy PowerSoil Extraction Kit (MoBio Laboratories, Carlsbad, CA, USA). DNA quality and content were checked using a NanoDrop spectrophotometer. The copy number of the bacterial community was determined by quantitative PCR (*q*PCR) using universal primers 515F and 907R (Biddle et al., 2008). Each sample was amplified in a 30 μl reaction mix containing 15 μL 2 × qPCR Mix, 0.5 μl forward primer (10 μM), 0.5 μl reverse primer (10 μM), and 2 μL template DNA. The *q*PCR conditions were: initial denaturation at 95°C for 10 min, followed by 40 cycles of 95°C for 30 s, 60°C for 30 s, 72°C for 30 s, and a final extension at 72°C for 10 min. The *q*PCR amplification was performed in triplicate with efficiencies >95% and *r*^2^ values >0.99.

The amplicon fragments using the same primer pairs were subjected to high-throughput sequencing on the Illumina MiSeq. Raw sequencing data were processed using the Quantitative Insights into Microbial Ecology (QIIME) pipeline (version 1.9.1). Sequences with masses less than 20 and lengths less than 300 bp were removed, matched to different samples based on specific tags, and assigned to separate files for the bacterial 16S *rRNA* gene. The remaining sequences were chimera removed and clustered into operational taxonomic units (OTUs) at 97% similarity using the Usearch algorithm. The representative sequence for each OTU with the highest abundance was then chosen. Taxonomic assignments were performed against the Silva database (version 138) for the bacteria community. The command “alpha_diversity.py” of the QIIME software was used to calculate the Shannon index of bacterial alpha-diversity after the rarefaction of all samples to the same sequencing depth of 28,883 sequences.

### Carbon use efficiency and microbial metabolic quotient

Soil microbial growth rate and respiration rate were determined by the ^18^O labeling method to determine carbon use efficiency (CUE) (Zheng et al., 2019). Briefly, 1 g of precultured soil sample was added into a 2 mL injection bottle, three replicates were set for each sample, and H_2_^18^O (97.0%, ABX, Israel) was added to one of them to label the soil with ^18^O. The abundance was adjusted to 20%, and an equal volume of deionized water was added to another portion as a natural ^18^O natural abundance control. After the added water was thoroughly mixed with the soil, the sample was placed in a 50 mL headspace vial, and aluminum caps were crimped on to seal the headspace vials. The headspace vial gas was then immediately replaced with CO_2_-free air to limit the CO_2_ concentration in the vial. A blank headspace vial without soil was used as a blank control throughout the experiment. After all manipulations, the incubation vials were incubated at 15°C for 24 h. After the incubation, the gas in the headspace vial was collected using a syringe with a three-way valve, and the CO_2_ concentration was measured using a gas chromatograph (GC-7890B, Agilent, USA). After freeze-drying the soil samples in the injection vials, the DNA was extracted and transferred to a silver cup, and then dried at 45°C. The ^18^O abundance and oxygen content were determined by an elemental analyzer coupled to an isotope ratio mass spectrometer using the EA-IRMS system (Thermo Scientific, TX, USA). The newly produced DNA content (*DNA_produced_*, μg) of the soil samples during the 24-h incubation period was calculated as follows:


$$DNA_{produced}=O_{total}\times \frac{at\%excess}{100\;}\times \frac{100}{at\%label\;}\times \frac{100}{31.21}$$

In the formula, *O_total_* (μg) is the oxygen content in the dried sample DNA; *at%_excess_* is the difference between the abundance of ^18^O in the labeled and unlabeled samples; *at%_label_* is the abundance of ^18^O in the final soil solution.

The ratio of MBC to DNA content of each precultured sample was calculated as a conversion factor (*fDNA*), and *fDNA* was used to convert the newly generated DNA content in the sample during the culture period into MBC content, thereby calculating the microbial growth rate (*Growth*, ng C· g^−1^ h^−1^) formula is as follows:


$$\mathrm{Growth}=\frac{\mathrm{fDNA}\times \mathrm{DNAproduced}\times 1000}{\mathrm{DW}\times t}$$

In the formula, *DW* (g) is soil dry weight; *t* (h) is cultivation time.

The calculation formula of microbial respiration rate (*Respiration*, ng C g^−1^ h^−1^) is as follows:


$$\mathrm{Respiration}=\frac{\Delta \mathrm{CO}2}{22.4\times \mathrm{DW}\times t\;}\times M\times V\times 1000\;$$

where *∆CO_2_* (ppm) is the increase in CO_2_ concentration in the closed headspace vessel during the incubation period of the soil sample; *M* is the molar mass of carbon (12.01 g mol^−1^); *V* (l) is the total volume of gas; *DW* (g) is the soil dry weight; *t* (h) is incubation time.

The formula for calculating the metabolic entropy (*q*CO_2_, ng C μg MBC^−1^ h^−1^) is as follows:


$$q\mathrm{CO}2=\frac{\mathrm{Respiration}}{\mathrm{MBC}}$$

The formula for calculating CUE is as follows:


$$\mathrm{CUE}=\frac{\mathrm{Growth}}{\mathrm{Growth}+\mathrm{Respiration}}$$

### Carbon mineralization

The determination of C mineralization adopts an indoor constant temperature culture, and C mineralization was determined by the alkali absorption technique: weigh 100 g of fresh soil in a 500 mL culture bottle and spread it on the bottom of the bottle. The water holding capacity is calculated when the maximum water holding capacity is 60%. Then, an absorption bottle containing 20 mL of NaOH solution at a concentration of 0.5 mol L^−1^ was placed in the culture bottle, the culture bottle was sealed with a cap, and placed in a constant temperature incubator at 25°C for 30 days under dark conditions. The solution in the absorption bottle was taken out, washed completely into the conical flask, and 20 mL of 1 mol L^−1^ BaCl_2_ solution and two drops of phenolphthalein indicator were added. The remaining NaOH was titrated and neutralized with 0.4 mol L^−1^ HCl solution until the red color disappeared, and the CO_2_ release by HCl consumption was calculated.


$$\begin{array}{c} \mathrm{Soil}\;C\;\mathrm{mineralization}\;\left(\mathrm{calculated}\;\mathrm{as}\;{\mathrm{CO}}_{2}\;\mathrm{release}\right)=c_{\mathrm{HCl}}\times \left(V_{0}-V_{1}\right)\\ \times 22/0.1, \end{array}$$

where *c*_HCl_ is the concentration of hydrochloric acid (mol l^−1^), *V*_0_ is the volume of the blank titration, and *V*_1_ is the volume of hydrochloric acid consumed.

### Nematode community and ecological indices

Baermann funnel method was used to extract nematodes (Cesarz et al., 2019). Briefly, 100 g of soil was spread evenly on the funnel device and then water was added to submerge the soil. The spring clip was opened to obtain an aqueous solution of nematodes after 72 h. The nematodes were killed at 60°C and stored in a 4% formalin solution. At least 150 nematodes per sample were collected and placed under a light microscope for nematode identification. Nematodes were classified into four trophic guilds based on morphology (Yeates, 1998), including bacterivores (Ba), fungivores (Fu), plant-parasites (Pp), and omnivores-predators (OP). Predation pressure of nematodes on bacteria was calculated as the ratio of the number of nematodes to bacterial copy number (Mathisen et al., 2016). Furthermore, we selected the nematode indices to indicate the food web status, including Shannon–Wiener index, channel index (CI), enrichment index (EI), structure index (SI), functional dispersion index (FDis), and free-living nematode maturity index (MI) (Ferris et al., 2001). The corresponding calculation formulas are as follows:


$$H=-\Sigma p_{i}\;\left(lnp_{i}\right)$$

$$MI=\Sigma \left(c-p_{i}\right)\;p_{i}\;\left(\mathrm{free}-\mathrm{living nematode}\right)$$

$$CI=100\;\left(0.8\times \mathrm{Fu2}/\left(3.2\times \mathrm{Ba1}+0.8\times \mathrm{Fu}\right)\right)$$

$$EI=100\times \left(e/\left(e+b\right)\right)$$

$$SI=100\times \left(s/\left(s+b\right)\right)$$

Predation pressure=Ba/bacterial copies

In these formulas, *p_i_* is the proportion of individuals of the ith taxon in the total number of nematodes; Ba, Fu, and PP represent the number of bacterivorous, fungivorous, and plant-parasitic nematodes, respectively; *e*, *b*, and *s* are enriched components (Ba1 and Fu2), basic components (Ba2 and Fu2), structural components (Ba3-Ba5, Fu3-Fu5, Om3-Om5, and Ca2-Ca5), respectively. The CI is between 0 and 100, greater than 50 indicates that the fungal degradation pathway is dominant, and less than 50 indicates that the bacterial degradation pathway is dominant. EI and SI vary from 0 to 100, with higher EI and SI indicating better nutritional status and better food web connectivity, respectively. Predation pressure refers to the predation pressure of bacterivorous nematodes on bacteria.

### Statistical analyses

Significant difference analysis of the data was performed using one-way ANOVA with SPSS 24.0 (Kim, 2017). Spearman’s rank correlation test was used to measure the correlations between soil properties, bacterial and nematode communities, and C mineralization (Sedgwick, 2014). The varpart function in the “vegan” package was used to determine the contribution of different types of factors to bacterial diversity, community composition, and C mineralization. Non-metric multidimensional scaling (NMDS) analysis based on Bray-Curtis distance was conducted to characterize differences in the structure of bacterial and nematode communities (Cox and Ferry, 1993). Redundancy analysis was used to estimate the relationships between soil variables, bacterial community, and nematode assemblage (Capblancq et al., 2018). The envfit function in the “vegan” package was used to perform a permutation test to test for potentially significant effects of soil properties and nematode index on community structure (Oksanen, 2015). The random forest model was constructed using the package “randomForest” of the R software (Liaw and Wiener, 2002). The “rfUtilities” and “rfPermute” packages were used to test the *p* value of each variable and the model, and to check the effect of each factor on the C mineralization. Structural equation modeling (SEM) was constructed to assess the potential effects of soil factors, and the bacterial and nematode communities on C mineralization. The R-packaging “lavan” was used for SEM analysis, and the data used in the model were standardized (Mamet et al., 2019).

## Results

### Soil chemical properties and carbon mineralization

**Figure 1:**
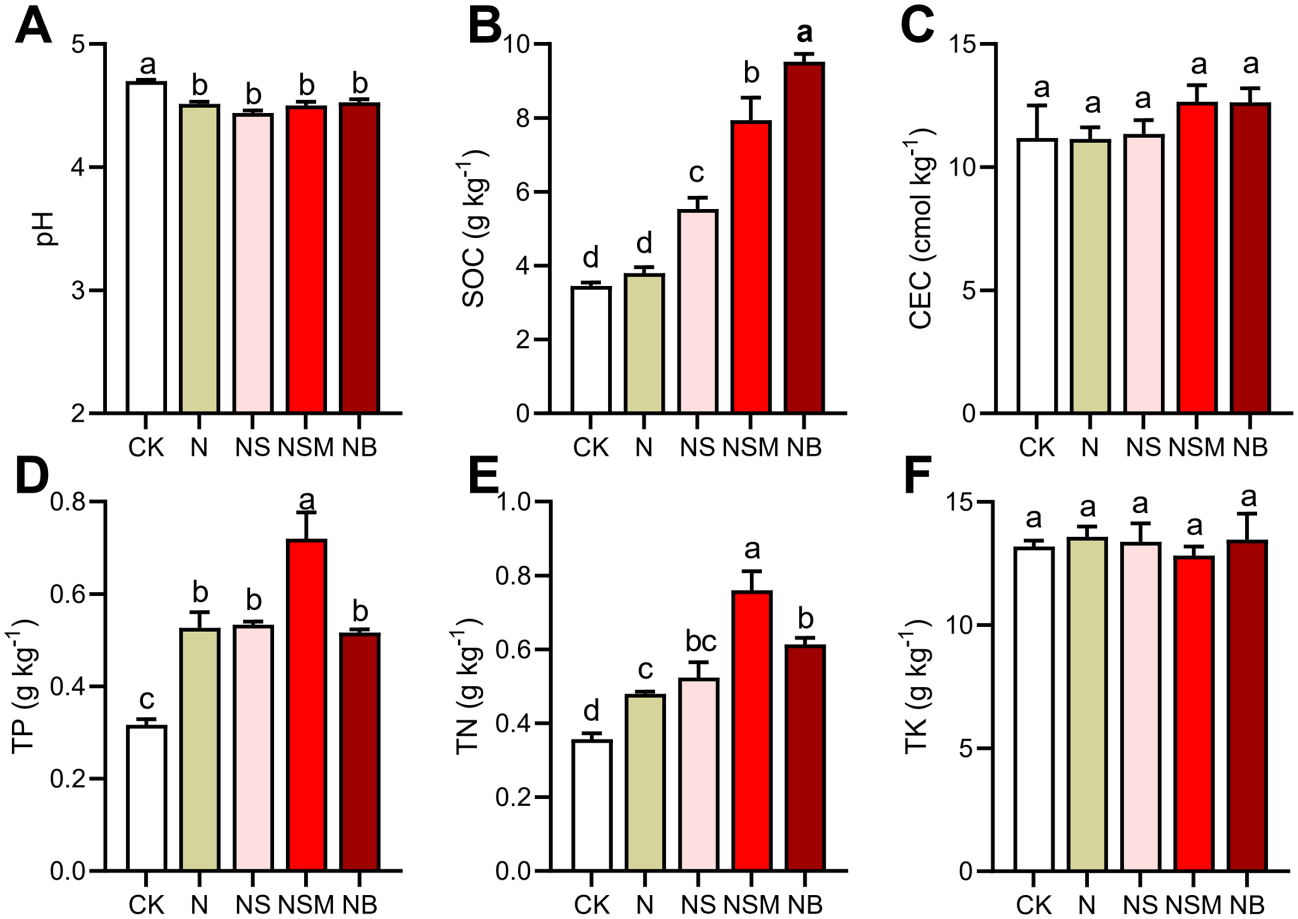
Soil chemical properties under the five fertilization treatments, including **(A)** pH, **(B)** soil organic carbon (SOC), **(C)** cation-exchange capacity (CEC), **(D)** total phosphorus (TP), **(E)** total nitrogen (TN), **(F)** total potassium (TK). Different lowercase letters indicate significant differences based on Tukey’s HSD test (*p* < 0.05). CK, no fertilizer; N, chemical fertilizers; NS, chemical fertilizers with straw; NSM, chemical fertilizers with straw and pig manure; NB, chemical fertilizers with biochar.

One-way ANOVA showed that soil chemical properties were significantly changed under different organic material amendments (Figure 1). Soil pH ranged from 4.4 to 4.8, and was significantly (*p* = 0.048) higher under NB treatment than under the other four treatments. Soil organic carbon (SOC) was significantly increased (*p* < 0.001) under NS, NSM, and NB treatments compared to CK and N treatments. Total phosphorus (TP) and total nitrogen (TN) were the highest under the NSM treatment and the lowest under CK treatment. There was no significant (*p* > 0.05) difference in cation-exchange capacity (CEC) and total potassium (TK) among all treatments. Carbon mineralization (CM) under NSM treatment was significantly (*p* < 0.05) higher than that under N, NS, and NB treatments (Supplementary Figure S1A). Carbon use efficiency (CUE) under N and NSM treatments was significantly (*p* < 0.05) improved compared to CK, NS, and NB treatments (Supplementary Figure S1B).

### Nematode community composition and ecological indices

**Figure 2:**
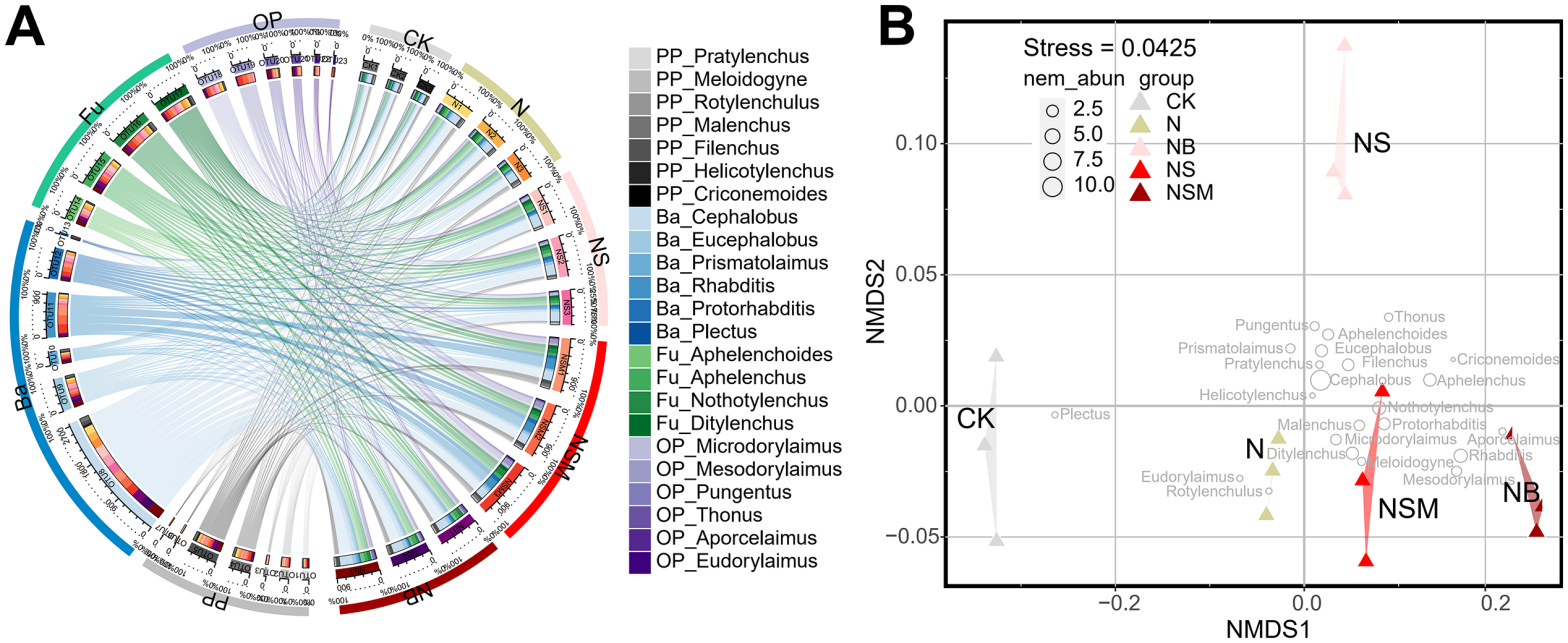
Chord diagram showing the relative abundances of nematode at the genus level under different fertilization treatments **(A)**. The non-metric multi-dimensional scaling plot (NMDS) depicts the distance between the samples based on Bray-Curtis similarities **(B)**. Different circle sizes represent the relative abundance of nematodes. CK, no fertilizer; N, chemical fertilizers; NS, chemical fertilizers with straw; NSM, chemical fertilizers with straw and pig manure; NB, chemical fertilizers with biochar.

**Figure 3:**
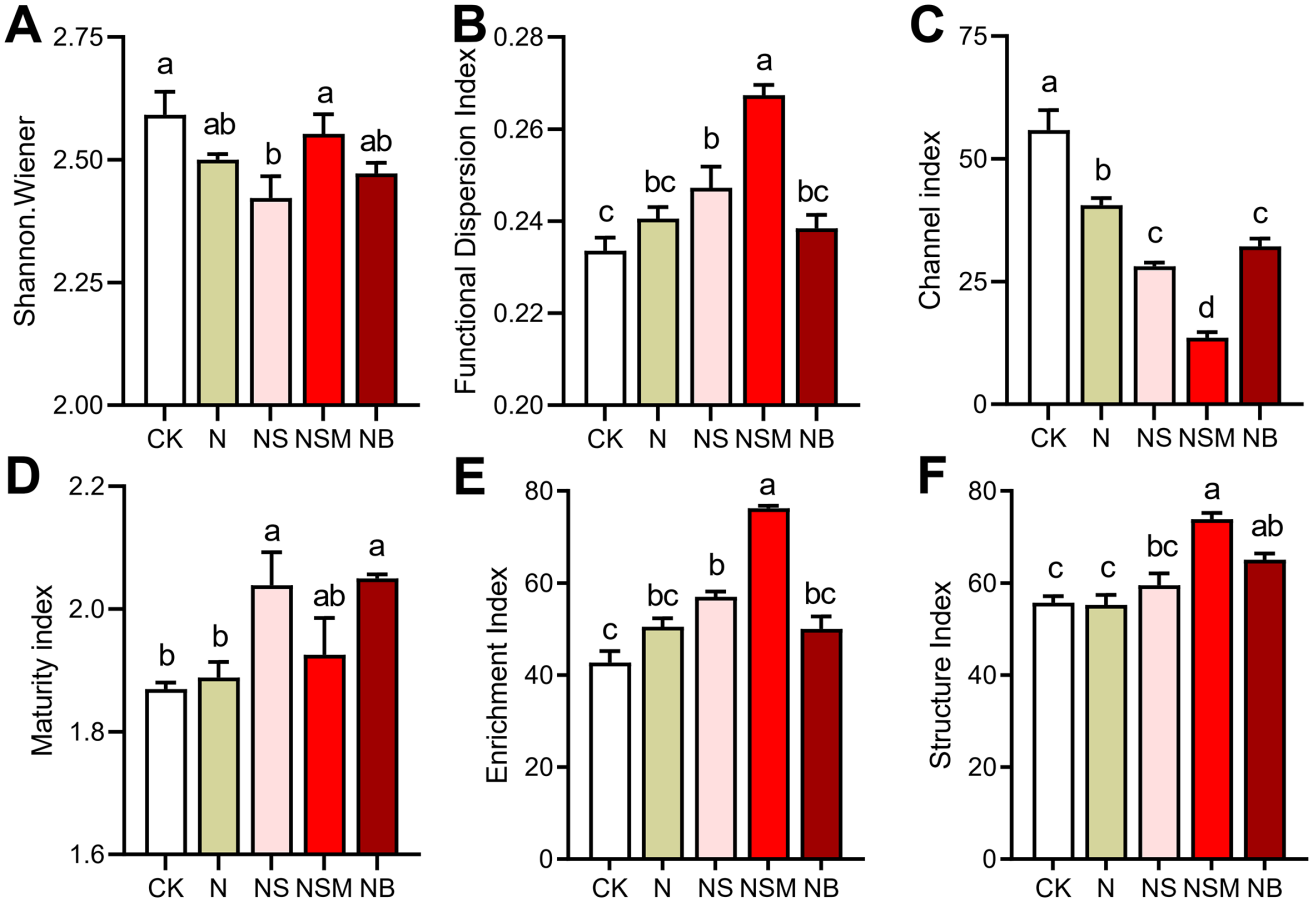
Nematode ecological index under the five fertilization treatments, including **(A)** Shannon–Wiener index, **(B)** functional dispersion index (FDis), **(C)** Channel index (CI), **(D)** maturity index (MI) of free-living nematodes, **(E)** Enrichment index (EI) and **(F)** Structure index (SI). Different lowercase letters indicate significant differences based on Tukey’s HSD test (*p* < 0.05). CK, no fertilizer; N, chemical fertilizers; NS, chemical fertilizers with straw; NSM, chemical fertilizers with straw and pig manure; NB, chemical fertilizers with biochar.

Among all treatments, the dominant trophic guilds of the nematode community were bacterivores (Ba, 47.6%), followed by fungivores (Fu, 25.7%), omnivores-predators (OP, 13.2%) and plant parasites (PP, 13.3%) (Figure 2A). The dominant genus of bacterivorous nematodes was *Cephalobus*, followed by *Eucephalobus* and *Protorhabditis* (Figure 2A). Nonmetric multidimensional scale (NMDS) analysis showed that the fertilization treatments significantly changed the composition of the nematode community (Figure 2B). Nematode community compositions showed that nematode assemblages under organic treatments were significantly (*p* < 0.05) distinct from that under CK treatment (Figure 2B). The numbers of total nematodes and bacterivores were significantly (*p* < 0.05) increased under NSM treatment than other treatments (Supplementary Figure S2A). Metabolic footprints of bacterivores and omnivores-predators were significantly (*p* < 0.05) increased under NSM treatment than N, NS, and NB treatments, while there was no significant difference in metabolic footprints of fungivores among different treatments (Supplementary Figure S2B). We further found that the ecological indices of the nematode community significantly (*p* < 0.05) varied among fertilization treatments. Nematode diversity, as indicated by the Shannon index, was significantly (*p* < 0.05) higher under NSM treatments than under N treatment (Figure 3A). Functional dispersion index (FDis) was significantly (*p* < 0.05) improved by NS and NSM treatments compared to CK treatment, while channel index (CI) followed the opposite trend (Figures 3B,C). The maturity index (MI) of free-living nematodes under NS and NB treatments was significantly (*p* < 0.05) higher than that under CK and N treatments (Figure 3D). The enrichment index (EI) and structure index (SI) were significantly (*p* < 0.05) higher under NSM treatment than under CK and N treatments (Figures 3E,F).

### Bacterial community and metabolic activities

**Figure 4:**
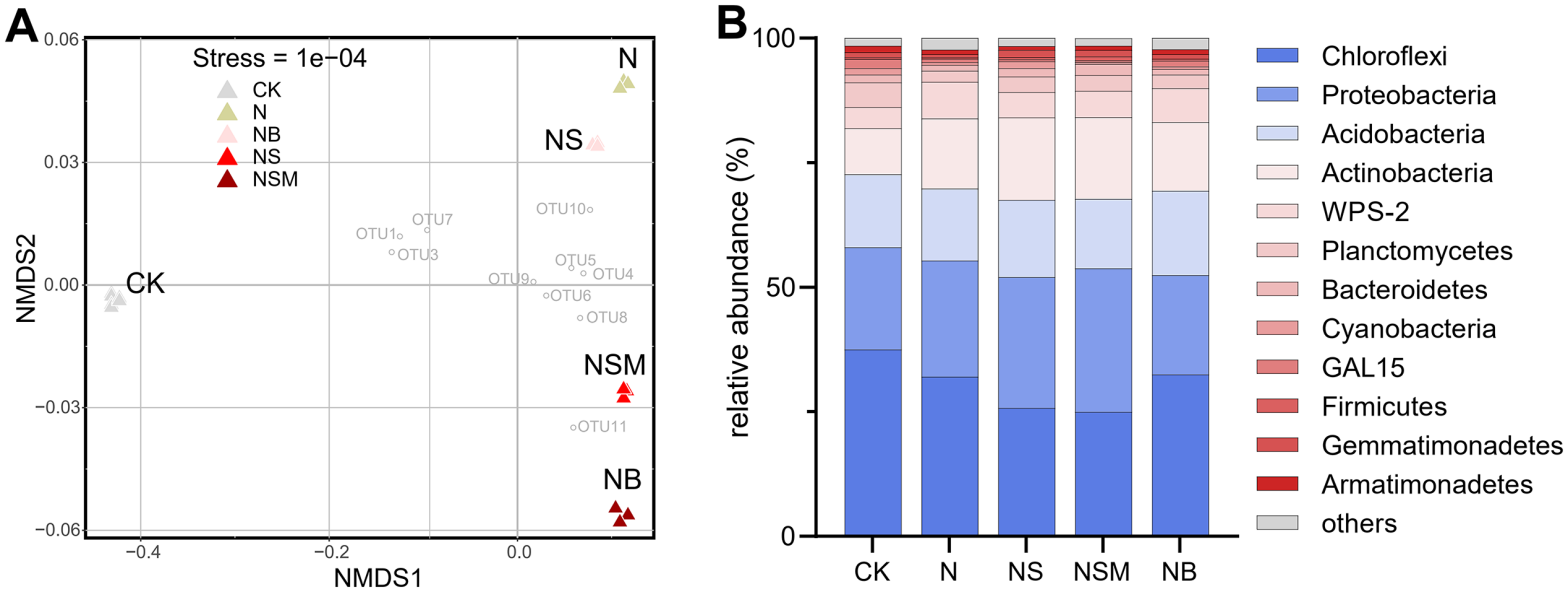
The non-metric multi-dimensional scaling plot (NMDS) of bacterial community structures of the five treatments **(A)**. The mean relative abundance of bacterial phyla within each treatment **(B)**. CK, no fertilizer; N, chemical fertilizers; NS, chemical fertilizers with straw; NSM, chemical fertilizers with straw and pig manure; NB, chemical fertilizers with biochar.

**Figure 5:**
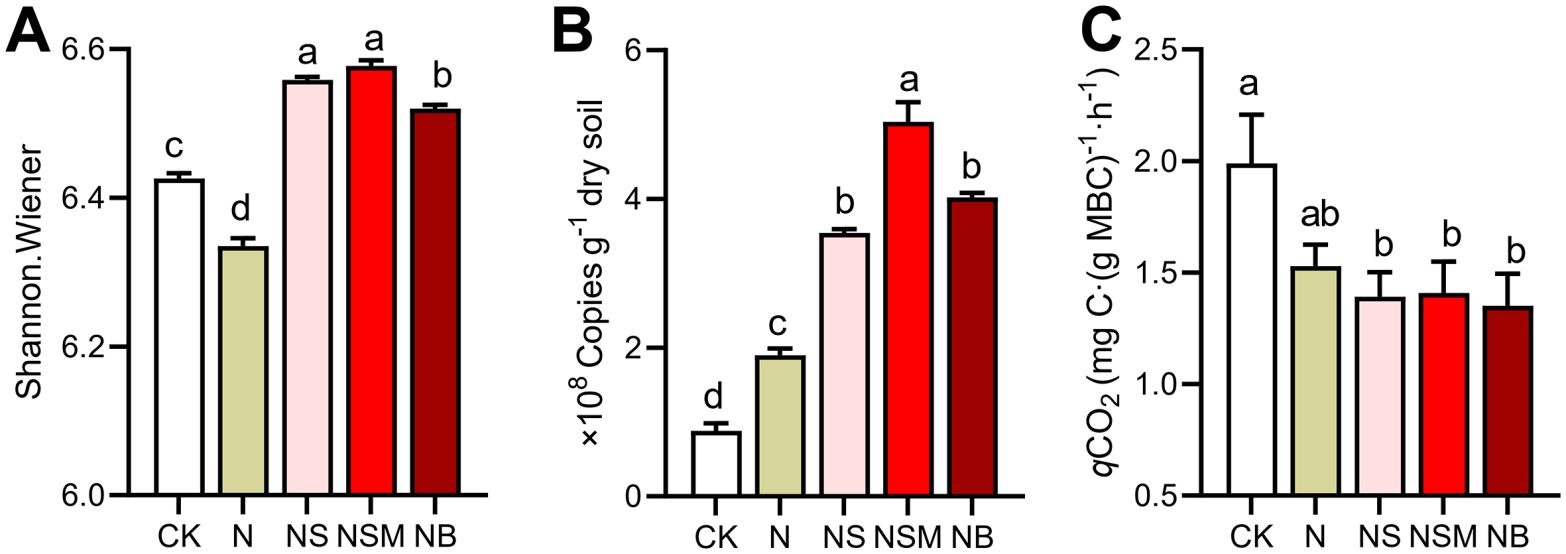
The diversity (**A**, Shannon index) and abundance (**B**, copy numbers of 16S *RNA*) of bacterial communities and microbial metabolic quotient (**C**, *q*CO_2_) under the five fertilization treatments. Different lowercase letters indicate significant differences based on Tukey’s HSD test (*p* < 0.05). CK, no fertilizer; N, chemical fertilizers; NS, chemical fertilizers with straw; NSM, chemical fertilizers with straw and pig manure; NB, chemical fertilizers with biochar.

Organic material amendments significantly (*p* < 0.05) differed the bacterial communities from those under CK and N treatments (Figure 4A). Bacterial community compositions showed that bacterial assemblages under four fertilization treatments were significantly (*p* < 0.05) distinct from that under CK treatment. At the phylum level, the bacterial community was dominated by Chloroflexi (30.5%), Proteobacteria (23.8%), Acidobacteria (15.1%), and Actinobacteria (14.0%) (Figure 4B). The relative abundance of Proteobacteria and Actinobacteria was significantly (*p* < 0.01) enriched under NS and NSM treatments compared to CK and NB treatments, while Chloroflexi and Acidobacteria were significantly higher under NB treatment (Figure 4B). The copy number and Shannon index of the bacterial community were significantly increased (*p* < 0.05) under NS, NSM, and NB treatments compared to CK and N treatments, while the microbial metabolic quotient (*q*CO_2_) followed the opposite trend (Figure 5).

**Figure 6:**
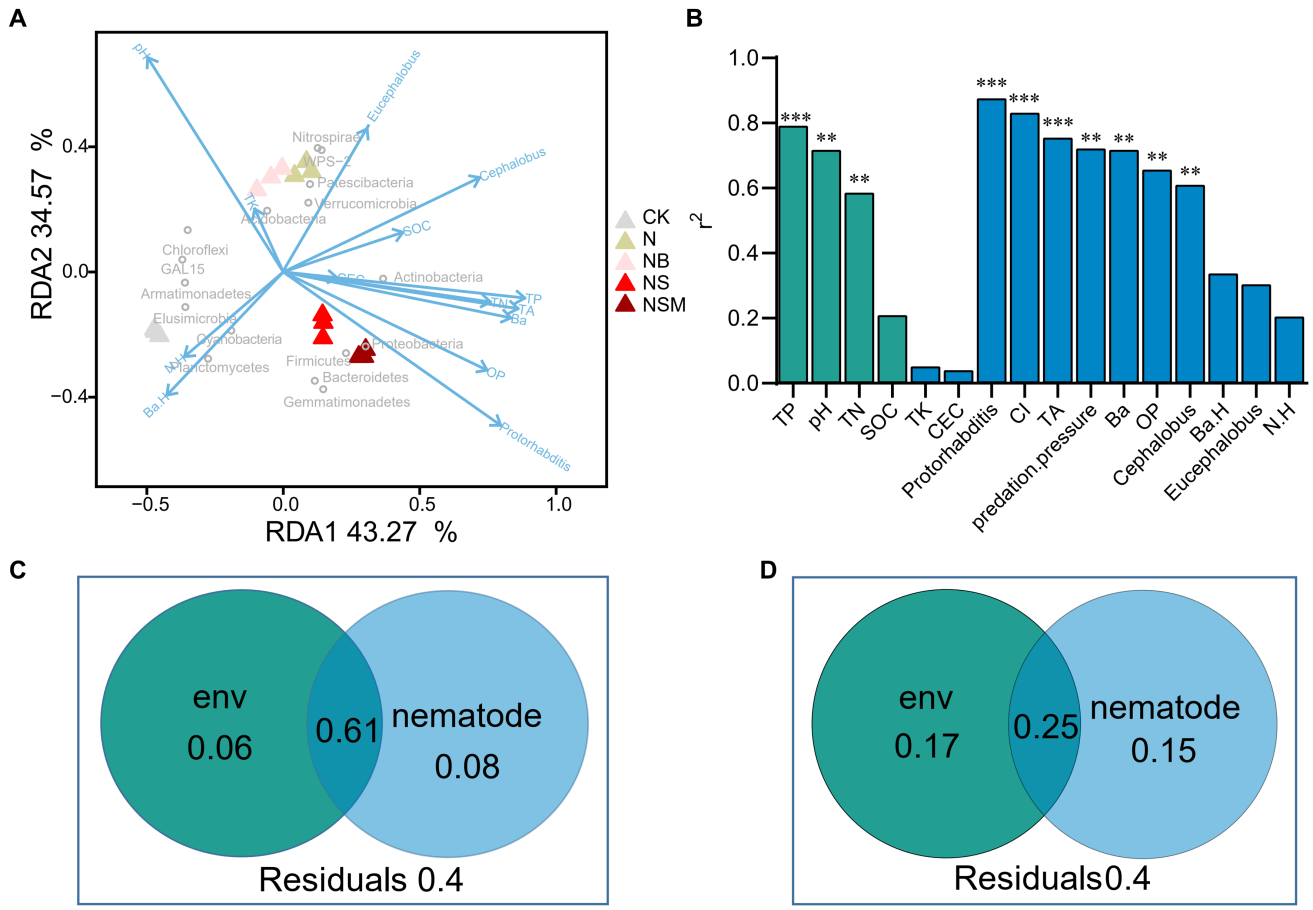
Redundancy analysis (RDA) indicates the relationships of soil properties and nematode indices with bacterial community **(A)**. Contributions of each factor to community structure based on envfit test (Monte Carlo permutation test) **(B)**. The proportion of soil chemical properties and nematodes explaining the composition (**C**, first principal coordinates, PCoA1) and abundance (**D**, copy number of 16S *rRNA*) of bacterial community. Soil chemical properties include soil pH, soil organic carbon (SOC), total nitrogen (TN), total phosphorus (TP), total potassium (TK), and cation-exchange capacity (CEC). The nematode community include the number of total nematodes (TA), bacterivores (Ba), and omnivores-predators (OP), and diversity (Shannon index, N.H). *p < 0.05, **p < 0.01, ***p < 0.001.

Redundancy analysis showed that all parameters explained 77.8% of the variation in the bacterial communities (Figure 6A). The main grouping factors under NS and NSM treatments were *Protorhabditis* and OP, and Proteobacteria and Actinobacteria were clustered with a high relative abundance of bacterivores and *Protorhabditis*. The envfit test indicated that soil chemical factors (TP, pH, and TN) and nematode assemblage (the numbers of total nematodes, bacterivores, omnivores-predators, *Protorhabditis* and *Cephalobus*, CI, and predation pressure) had significant effects on the bacterial community composition (*r*^2^ = 0.61 to 0.87, *p* < 0.05) (Figure 6B). Variance partitioning analyses showed that the interactions between the environmental factors and nematode indices could explain 61% of the variation of bacterial communities, while the two independent explanations were only 6 and 8% (Figure 6C). Environmental factors and nematode indices can independently explain 15 and 17% of the variation of bacterial biomass, respectively, and interactions between the two major components were up to 25% (Figure 6D).

### Potential nematode-bacteria interactions affected C sequestration

**Figure 7:**
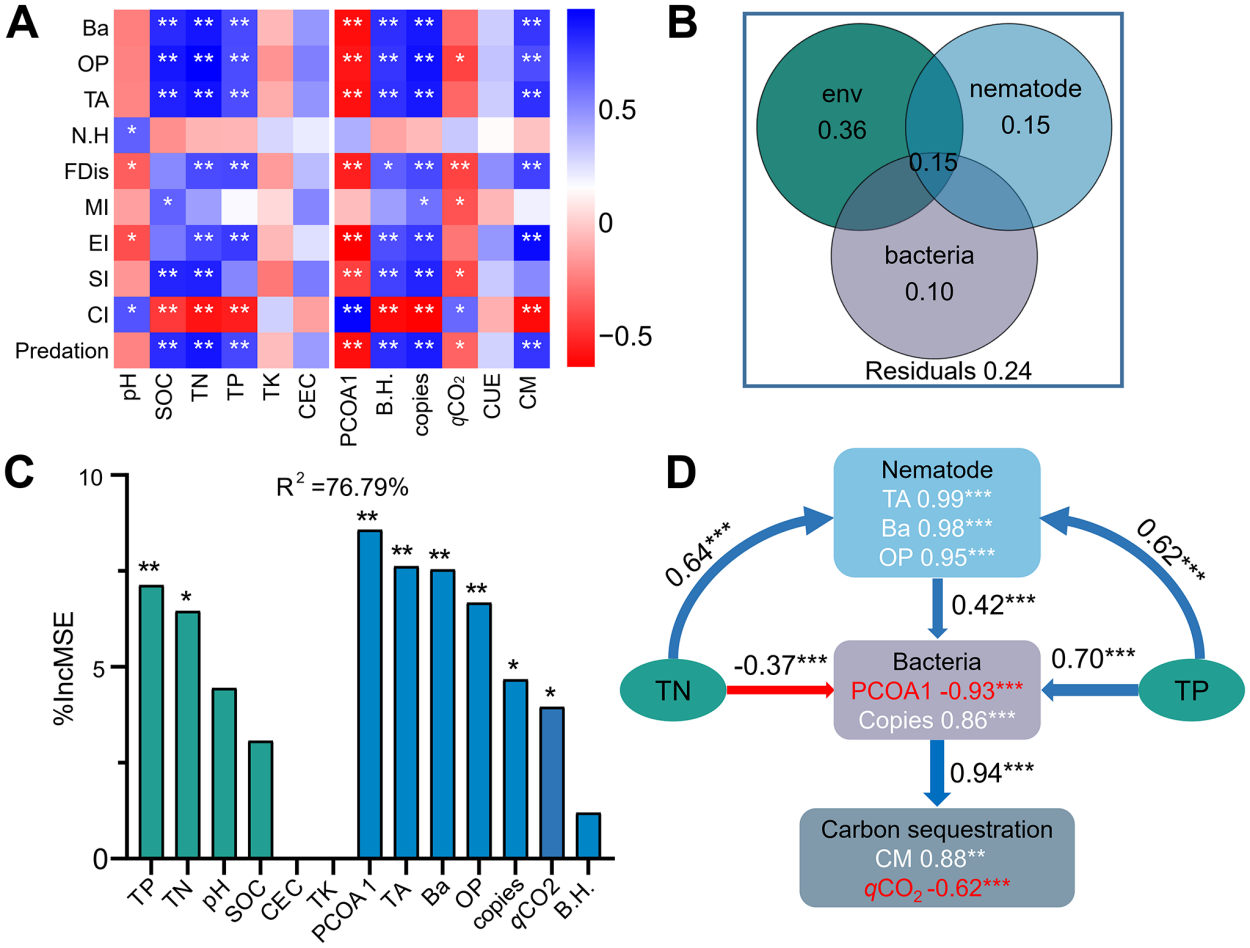
Spearman’s correlation coefficients between soil properties, the bacterial and nematode communities, and carbon mineralization **(A)**. Blue and red denote positive and negative correlations, respectively. * *p* < 0.05, ** *p* < 0.01, *** *p* < 0.001. Contribution of soil chemical properties and the bacterial and nematode communities to carbon mineralization using variance partitioning analysis **(B)**. Mean predictor importance (% of increased mean square error, MSE) of soil chemical properties and the bacterial and nematode communities on carbon mineralization using random forest modeling **(C)**. Structural equation modeling shows the direct and indirect effects of soil properties and the bacterial and nematode communities on carbon sequestration **(D)**. Soil chemical properties include soil pH, soil organic carbon (SOC), total nitrogen (TN), total phosphorus (TP), total potassium (TK), and cation-exchange capacity (CEC). The bacterial community include diversity (Shannon index, B.H), abundance (copy number of 16S rRNA) and composition (first principal coordinates, PCoA1). The nematode community include the number of total nematodes (TA), bacterivores (Ba), and omnivores-predators (OP), and diversity (Shannon index, N.H), Channel index (CI), Enrichment Index (EI) Structure Index (SI), functional dispersion index (FDis), and free-living nematode maturity index (MI) Carbon sequestration is indicated by carbon mineralization (CM) and microbial metabolic quotient (*q*CO_2_).

Correlation heatmap analysis showed that the bacterial diversity and abundance and CM were significantly positively correlated with the numbers of total, bacterivorous and omnivorous-predatory nematodes, predation pressure, EI, and FDis (*r* = 0.66 to 0.89, *p* < 0.05), but negatively correlated with CI (*r* = −0.84 to −0.87, *p* < 0.05) (Figure 7A). In contrast, the bacterial community composition (PCOA1) (*r* = −0.76 to −0.83, *p* < 0.05) and *q*CO_2_ (*r* = −0.50 to −0.64, *p* < 0.06) exhibited negative relationships with the abundance of total and bacterivorous nematodes, predation pressure, SI, and FDis. Variance partitioning analyses showed that soil chemical factors, nematode indices, and bacterial characteristics individually explained 36, 15, and 10% of the variances in CM, and interactively contributed to 15% of the observed variation (Figure 7B). Specifically, random forest analysis soil TN (6.5%), TP (7.1%), the abundance (4.7%) and composition (8.6%) of the bacterial community, and the numbers of total nematode (7.6%), bacterivores (7.5%), and omnivores-predators (6.7%) contributed significantly (*p* < 0.05) to C mineralization (Figure 7C). Structural equation modeling indicated that the nematode assemblage indirectly affected soil C sequestration by regulating bacterial community structure and biomass (Figure 7D). Specifically, the bacterial community was positively associated with the nematode assemblage (*r* = 0.42, *p* < 0.001), but negatively associated with *q*CO_2_ (*r* = −0.94, *p* < 0.001).

## Discussion

### Bacterial community varied with organic material amendments

We revealed that organic material amendments could significantly promote soil fertility by improving SOC, TN, and TP. Corn straw and pig manure are rich in soil organic matter and nutrients, and biochar is a carbon-rich material. The addition of these organic materials greatly increases SOC content (Cooper et al., 2011; Sandhu et al., 2017). Notably, SOC, TP, and TN were significantly increased under NSM treatment compared to N treatment, but not significantly increased under NS treatment. The simultaneous utilization of straw and manure reduces the C/N ratio of straw, resulting in faster decomposition and turnover rates of soil nutrients (Zheng et al., 2022a). Compared with corn straw, manure treatments can substantially enhance soil phosphorus content due to the high proportion of phosphorus. The NSM treatment significantly increased the contents of TN and TP by improving the organic N mineralization of straw compared with NS and NB treatments. In particular, the more reasonable ratio of corn straw and pig manure leads to a higher efficiency of the positive priming effect on straw decomposition, and a higher level of soil fertility (Abdalla et al., 2022). Soils treated with straw and pig manure enrich the food resources for microbial growth, and stimulate bacterial biomass and diversity (Hussain et al., 2019). Biochar with a good porous structure may provide habitats for microorganisms that are protected from predators, thereby preserving bacterial biomass within the biochar (Pietikäinen et al., 2000). Compared to NB treatment, we found that lower SOC under NSM treatment was not consistent with the trend of bacterial abundance, which may be partially explained by the alleviation of nutrient limitation (Wei et al., 2020). Organic material amendments promote soil structural complexity and heterogeneity, and better soil quality contributes to biodiversity by increasing spatial niches (Bebber and Richards, 2022). Soils with more abundant resources and higher nutrient availability can support higher bacterial diversity (Clarke and Gaston, 2006).

### Nematode community affected by organic material amendments

We observed that the numbers of total nematodes, bacterivores, and omnivores-predators were considerably increased under NS, NSM, and NB treatments due to the improved food sources and better habitats. Organic material amendments not only create better habitats for nematodes and facilitate their movement through soil pore water, but also improve the ability of nematodes to capture abundant food resources (bacteria) due to the increased soil organic matter (Liu et al., 2016; Jiang et al., 2018b). In addition, our results determined that fertilization practices increased the metabolic footprint of four nematode groups, suggesting that more carbon flowed into the soil nematode community for nematode reproduction (Zhang et al., 2016). Straw and manure amendments increased the functional dispersion index (FDis), indicating weaker species competition within a community and more complete utilization of available resources (Prada-Salcedo et al., 2021). Organic material amendments result in a more bacterially dominated fast cycle decomposition pathway, as indicated by the high number of bacterial-feeding nematodes and low CI value (Zhong et al., 2017). The interaction between EI and SI has been widely used to indicate resource enrichment and food web status (Ferris et al., 2001). The higher values of EI and SI under NSM treatment represent better nutrient status and a more complex soil food web. The higher MI value supported that the food web was more mature under the NS and NB treatments, whereas the lower MI value under NSM treatment was mainly due to the increased presence of opportunistic bacterivorous nematodes. For example, *Rhabditis* and *Protorhabditis* are capable of rapidly reproducing and responding to bacteria when food resources are abundant at high nutrient levels (Hu and Qi, 2010).

### Nematode-bacteria interactions enhanced C sequestration

Microbial metabolic quotient (*q*CO_2_) and carbon use efficiency (CUE) are commonly indicated as the rate of C storage in diverse ecosystems, with lower *q*CO_2_ reflecting better soil biophysical conditions and higher CUE reflecting higher metabolic efficiency (Chavarria et al., 2018; Feng et al., 2021). We suggested that the lower *q*CO_2_ value probably indicates better soil biophysical conditions caused by the organic material amendments (NS, NSM, and NB treatments). The variation of *q*CO_2_ may also be related to microbial community composition and biomass, and the values of lower *q*CO_2_ and higher CUE indicate a higher C sequestration potential (Ros et al., 2006; Liu et al., 2020). Phosphorus (P) limitation may have a greater impact on CUE in red soils (Feng et al., 2021). Since biochar is a reluctant compound with extremely limited P content, bacteria need to secrete more extracellular enzymes to acquire nutrients, resulting in a lower CUE value (Allison, 2005; Spohn, 2016). Biochar amendment can induce competition with potential keystone taxa to promote the abundance and diversity of the bacterial community, thereby reducing carbohydrate catabolism and *q*CO_2_ (Chen et al., 2019).

Soil bacterial community plays a crucial role in organic matter decomposition and nutrient availability (Steenwerth et al., 2002). Our results indicated that the nematode predation may increase the abundance of the bacterial community, with positive cascading effects on C sequestration. Numerous studies have pointed out that the bacterial community composition and biomass can mediate *q*CO_2_ and CUE, leading to variations in C mineralization processes (Spohn and Chodak, 2015; Fanin and Bertrand, 2016). Although the higher bacterial abundance indicates that more microorganisms are involved in the C decomposition, *q*CO_2_ was generally found to decrease with increasing microbial biomass. Nematodes are thought to be functionally critical in microbial food webs. Bacterivorous nematodes are primarily involved in regulating rates of C mineralization and altering pathways of energy flow and nutrient cycling by affecting the abundance and composition of the bacterial community (Hungate et al., 2021; Tian et al., 2022). We found that the bacterial biomass significantly increased with the increased predation pressure, with the highest biomass under NSM treatment. Trophic dynamics suggest that the prey biomass at a given trophic level is maximal at a moderate level of predation (Carpenter et al., 1985). Bacterivores can positively influence the diversity and abundance of the bacterial community by providing new niches for colonization and by creating physical and behavioral refuges for prey (Cressman and Garay, 2009; Zheng et al., 2022b). Selective predation by bacterivorous nematodes alters the bacterial community, specifically promoting the relative abundance of Proteobacteria to alter C mineralization (Neidig et al., 2011). *Protorhabditis* shows a strong effect on Gram-negative bacteria (e.g., Proteobacteria), which may be related to their thinner cell wall that makes them more easily digestible by predators (Wilson, 1993). In this case, nematode-bacteria interactions significantly increase the metabolic capacities of six substrate groups of carbon sources, including carbohydrates, carboxylic acids, amino acids, polymers, phenolic acids, and amines (Jiang et al., 2018b). In addition, bacterivore predation further shows a positive relationship with the size and turnover rate of C pools and contributes positively to C sequestration (Zhang et al., 2013; Jiang et al., 2018a). The C sequestration efficiency was improved under NSM treatment, which could be further explained by the bacterial characteristics and nematode indices. The higher level of SOC and TP implied sufficient nutrient resources, which was supported by the higher EI value under NSM treatment than under CK treatment. However, the lower CI indicates the superiority in ensuring less carbon flux to higher trophic levels and the degradation of more labile carbon in the bacterial decomposition channel (Tabarant et al., 2011).

## Conclusion

Our findings indicated that organic material amendments enhanced the abundance of bacterial and nematode communities, and significantly altered the structure of bacterial and nematode communities. The higher values of EI, SI, and MI indicated a more mature and stable food web structure, while the low value of CI indicated dominant bacterial decomposition pathways under organic material amendments. We found that nematode predation could increase bacterial abundance and enhance C sequestration, thereby facilitating C accumulation and storage. Taken together, our research integrates the ecology of nematode assemblages with empirical evidence to provide insights into nematode predation driving C dynamics under organic material amendments. As such, a comprehensive understanding of the biological mechanisms of C sequestration may have important implications for the development of sustainable agroecosystems.

## Data availability statement

The datasets presented in this study can be found in online repositories. The names of the repository/repositories and accession number(s) can be found in the article/Supplementary material.

## Author contributions

YJ and BS designed all the experiments. GS, LL, and YJ wrote the manuscript. GS, GZ, ZZ, JZ, and YS were responsible for performing the field and lab experiments. All authors analyzed all data, discussed the results, critically reviewed the manuscript, and approved its publication. All authors read and approved the final manuscript.

## Funding

This study was supported by the National Key Research and Development Program (2022YFD1900603), National Natural Science Foundation of China (41922048, 42177298, and 42107336), Jiangxi Provincial Natural Science Foundation (20224BAB215054), the Youth Innovation Promotion Association of CAS (Y2021084), Double Thousand Plan of Jiangxi Province, and the Science and Technology Project of Jiangsu Province (BE2022393).

## Conflict of interest

The authors declare that the research was conducted in the absence of any commercial or financial relationships that could be construed as a potential conflict of interest.

## Publisher’s note

All claims expressed in this article are solely those of the authors and do not necessarily represent those of their affiliated organizations, or those of the publisher, the editors and the reviewers. Any product that may be evaluated in this article, or claim that may be made by its manufacturer, is not guaranteed or endorsed by the publisher.
