# Supplementary material for: Interaction between nematodes and bacteria enhances soil carbon sequestration under organic material amendments
Source: Front Microbiol. 2023 May 12;14:1155088. doi: 10.3389/fmicb.2023.1155088 (PMC10213412; doi:10.3389/fmicb.2023.1155088)
Supplement: Supplementary file 1 [file Data_Sheet_1.docx]

**Supplementary Materials for**

**Interaction between nematodes and bacteria enhances soil carbon sequestration under organic material amendments**

Guangping Shi, Luan Lu, Guofan Zhu, Zhaoyang Zeng, Jie Zheng, Yue Shi, Bo Sun, Yuji Jiang*

*** Corresponding authors:**

Yuji Jiang yjjiang@issas.ac.cn

**This PDF file includes:**

Figures S1 to S2


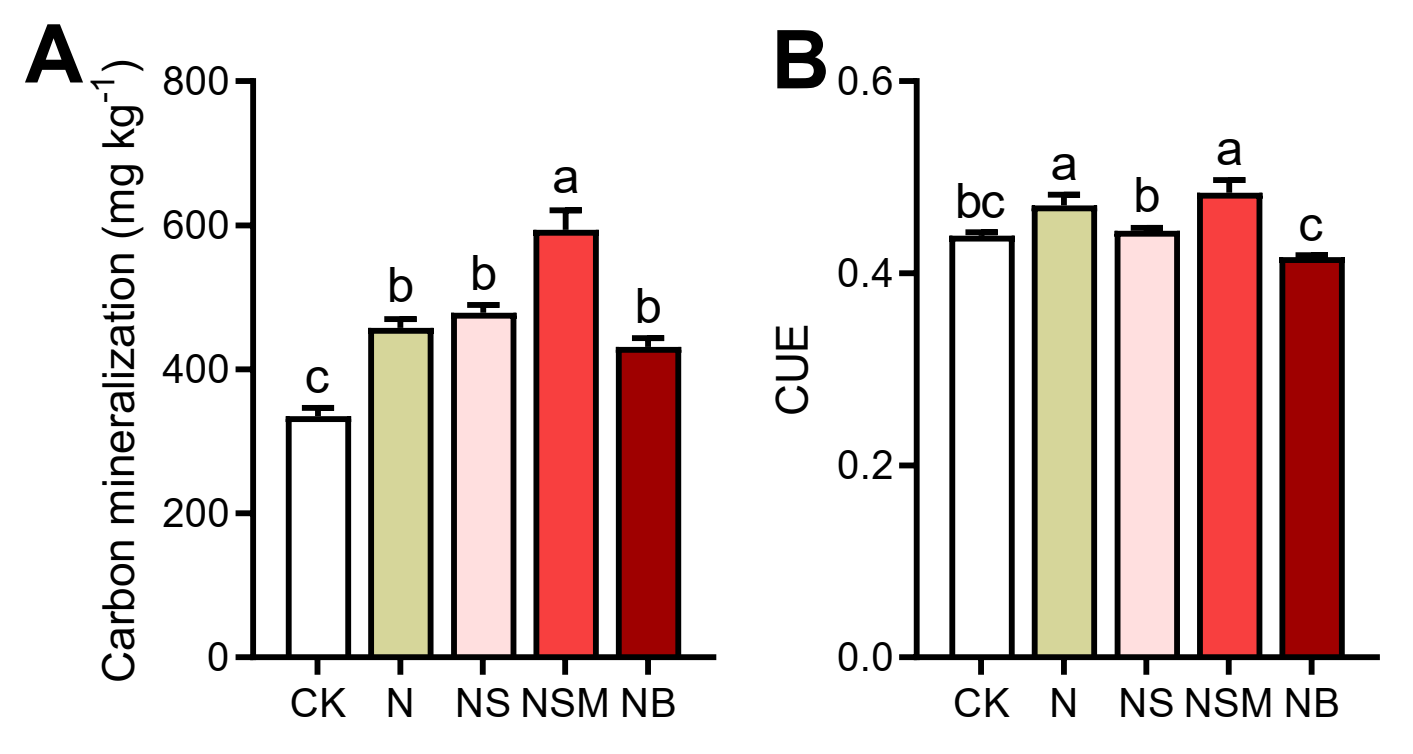


**FIGURE S1** Carbon mineralization (A) and microbial carbon use efficiency (B, CUE) under the five fertilization treatments. Different lowercase letters indicate significant differences based on Tukey’s HSD test (*P* < 0.05). CK, no fertilizer; N, chemical fertilizers; NS, chemical fertilizers with straw; NSM, chemical fertilizers with straw and pig manure; NB, chemical fertilizers with biochar.


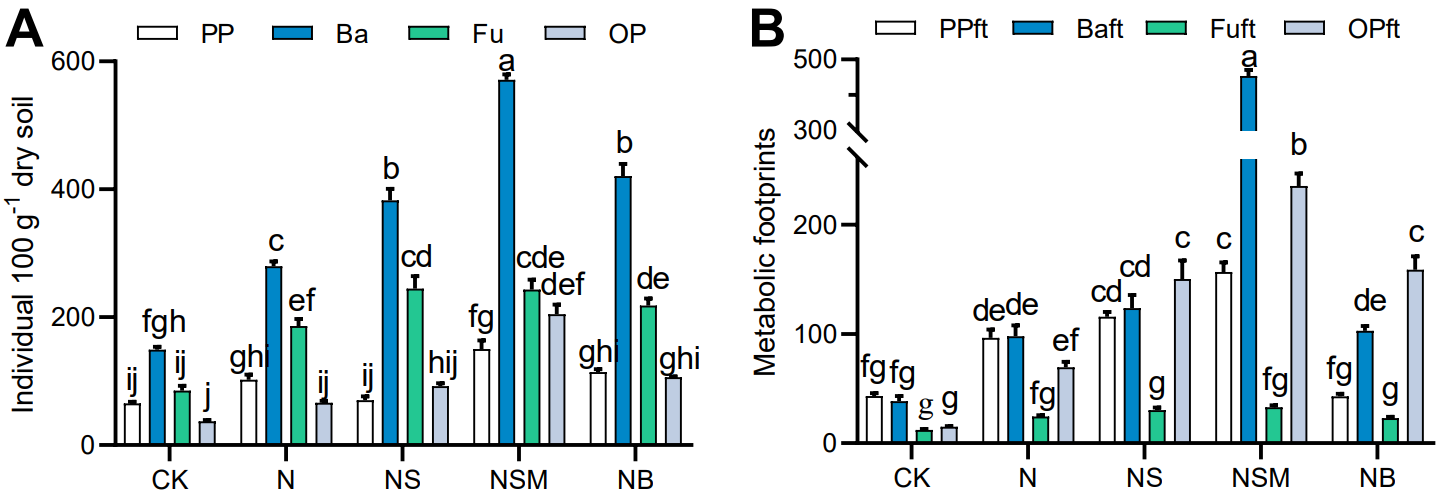


**FIGURE S2** Distribution of different nematode trophic guilds (A) and metabolic footprints (B) under different treatments. Different lowercase letters indicate significant differences based on Tukey’s HSD test (*P* < 0.05). Baft, metabolic footprints of bacterivores (Ba); Fuft, metabolic footprints of fungivores (Fu); PPft: metabolic footprints of plant parasites (Pp); OPft, metabolic footprints of omnivores-predators (OP). CK, no fertilizer; N, chemical fertilizers; NS, chemical fertilizers with straw; NSM, chemical fertilizers with straw and pig manure; NB, chemical fertilizers with biochar.
